# *N*-(2,2-Dimethyl-1-(quinolin-2-yl)propylidene) arylaminonickel Complexes and Their Ethylene Oligomerization

**DOI:** 10.3390/molecules22040630

**Published:** 2017-04-13

**Authors:** Hongyi Suo, Tong Zhao, Yiqing Wang, Qing Ban, Wen-Hua Sun

**Affiliations:** 1 Key Laboratory of Engineering Plastics and Beijing National Laboratory for Molecular Sciences, Institute of Chemistry, Chinese Academy of Sciences, Beijing 100190, China; suohongyi@iccas.ac.cn (H.S.); wyqing2015@126.com (Y.W.); 2 CAS Research/Education Center for Excellence in Molecular Sciences, University of Chinese Academy of Sciences, Beijing 100049, China; 3 School of Materials Science and Engineering, Qilu University of Technology, Jinan 250353, China; zhaotong1218@163.com

**Keywords:** nickel complex, *N*-(2,2-dimethyl-1-(quinolin-2-yl)propylidene) arylamine, ethylene oligomerization, schiff bases

## Abstract

A series of *N*-(2,2-dimethyl-1-(quinolin-2-yl)propylidene) arylamines was sophisticatedly synthesized and reacted with nickel(II) bromine for the formation of the corresponding nickel complexes. All the organic compounds were characterized by IR, NMR spectra and elemental analysis, while all the nickel complexes were characterized by IR spectra and elemental analysis. On activation with ethylaluminium sesquichloride (EASC) and modified methylaluminoxane (MMAO), all nickel precatalysts exhibited good activities toward ethylene oligomerization, indicating the positive efficiency of gem-dimethyl substitutents; in which major hexenes were obtained with MMAO. The catalytic parameters were verified, and the steric and electronic influences of substituents with ligands were observed, with a slight change of activities under different ethylene pressures.

## 1. Introduction

**Figure 1:**
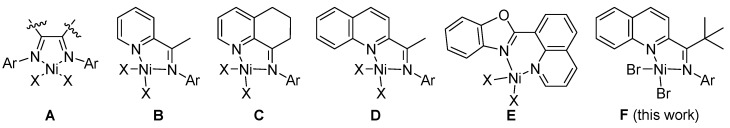
Representative examples of geometry-constrained iminopyridylmetal precatalysts.

The discovery of diiminonickel halides (**A**, Figure 1) as high active precatalysts in ethylene reactivity symbolized a milestone of late-transition metal catalytic systems [1], and resurrected nickel chemistry in the petrochemical industry with regard to the Shell Higher Olefin Process (SHOP) using a nickel catalyst [2]. The fast development of nickel precatalysts have been reviewed in a number of reviews [3,4,5]. To enhance the catalytic performances of nickel precatalysts, one method has modified diimino-ligands with different substituents, especially bulkier ones [6,7,8,9,10], whilst another approach has designed alternative nickel precatalysts of model ligands, such as 2-(1-aryliminoethyl)pyridines (**B**, Figure 1) [11,12,13,14,15], 8-(1-aryliminoethyl)-5,6,7-trihydroquinolines (**C**, Figure 1) [16,17,18,19,20], 2-(1-aryliminoethyl) quinolines (**D**, Figure 1) [21], 8-benzothiazolylquinolines (**E**, Figure 1) [22] and tridentate 2-imino-1,10-phenanthrolines [23]. Principally, models of **A** [1,6,7,8,9,10], **B** [11,12,13,14,15] and **C** [16,17,18,19,20] are potential precatalysts toward ethylene polymerization; we extensively investigated and modified **C** through controlling the fused-ring numbers in five [24] and seven [25,26] within their ligands, tailoring the resultant polyethylenes. There are few chances to tune models of **D** [21] and **E** [22]. However, encouraged with success of the *gem*-dimethylation for strained ligands [20,24], the 2,2-dimethyl-1-(quinolin-2-yl) propan-1-one was prepared and used to form *N*-(2,2-dimethyl-1-(quinolin-2-yl)propylidene) arylamines and their nickel complexes (**F**, Figure 1). The title complexes exhibited high activities toward ethylene oligomerization, producing hexenes as the major product. Herein the synthesis and characterization of the title complexes are reported along with their ethylene oligomerization.

## 2. Results

### 2.1. Synthesis and Characterization of Ligands and Nickel Complexes

**Scheme 1:**
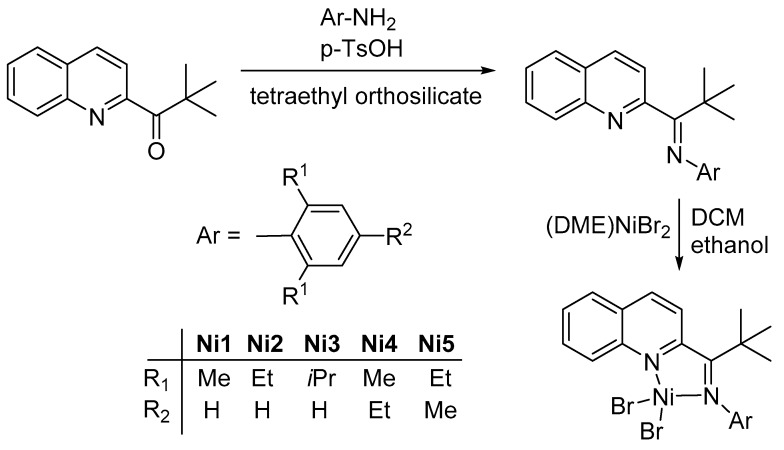
Synthetic procedure for **L1**–**L5** and **Ni1**–**Ni5**.

A series of *N*-(2,2-dimethyl-1-(quinolin-2-yl)propylidene) arylimines (**L1**–**L5**) was prepared in moderate yields by the Schiff base condensation of 2,2-dimethyl-1-(quinolin-2-yl)propan-1-one with anilines in the presence of a catalytic amount of p-toluenesulfonic acid. All of the organic compounds were routinely characterized by ^1^H- and ^13^C-NMR, FT-IR spectra, as well as elemental analysis. The addition of a 0.9 equivilent of (DME) NiBr_2_ (DME = 1,2-dimethoxyethane) to a mixture solution involving dichloromethane and ethanol of the prepared ligands, respectively, resulted in the formation of the correspondent nickel complexes (**Ni1**–**Ni5**) in good yield (Scheme 1). All of the nickel complexes were characterized by FT-IR spectra and elemental analysis.

According to the FT-IR spectra, the stretching vibrations of the ν_(C=N)_ in the abovementioned nickel complexes shifted to lower values (1613–1629 cm^−1^) with weaker intensity compared to those of the corresponding free ligands (1633–1652 cm^−1^), indicating the effective coordination between the sp^2^-nitrogen and the nickel atom.

### 2.2. Ethylene Oligomerization

In order to determine the most efficient co-catalyst for use in ethylene polymerization studies, as inspired by previous experiences of the [Al]/[Ni] molar ratios and reaction temperature [16,17,18,19,20], complex **Ni1** was explored in toluene at 30 °C with various co-catalysts such as methylaluminoxane (MAO), modified methylaluminoxane (MMAO), ethylaluminium sesquichloride (EASC, Et_3_Al_2_Cl_2_), diethylaluminium chloride (Et_2_AlCl) and triethylaluminium (Et_3_Al), respectively. Their results are tabulated in Table 1. Low activity was observed with both Et_3_Al or MAO, producing mainly butenes. With the other three co-catalysts, the systems exhibited high activity in the range of 1.07 × 10^6^–1.33 × 10^6^ g mol^−1^ (Ni) h^−1^. It is worth noting that there was a lower amount of butene observed in the use of MMAO and EASC, especially indicating major hexenes in the system with MMAO. Therefore, further studies were conducted with MMAO and EASC as co-catalysts.

#### 2.2.1. Ethylene Oligomerization in Presence of EASC

The reaction parameters greatly affect the catalytic activity and, using EASC as a co-catalyst, we determined the optimal conditions of the oligomerization containing the Al/Ni molar ratio, reaction temperature and run time. The results are collected in Table 2. Herein, **Ni1** was typically screened for ethylene oligomerization reaction parameters at 5 atm ethylene pressure.

Firstly, with the temperature at 30 °C and the run time set at 30 min, increasing the Al/Ni molar ratio from 200 to 500 resulted in an increase trend, with the highest activity of 2.17 × 10^6^ g mol^−1^ (Ni) h^−1^ observed at an Al/Ni molar ratio of 500 (entries 1–4, Table 2). Subsequently, as the molar ratio still aggrandized, the activity gradually decreased down to 1.11 × 10^6^ g mol^−1^ (Ni) h^−1^ (entries 5–7, Table 2). This suggested that the enlargement of the Al/Ni molar ratio results in the rate of chain termination exceeding the rate of chain propagation, thus forming longer chain oligomers.

Next, with the Al/Ni molar ratio fixed at 500 with 30 min, the activity for ethylene oligomerization was substantially affected by the changes of reaction temperature (entries 4 and 8–11, Table 2). On increasing the temperature from 20 °C to 60 °C, the activity was initially increased to a summit with 3.34 × 10^6^ g mol^−1^ (Ni) h^−1^ observed at 40 °C, and the value subsequently went down. This drop in catalytic performance along with the increasing temperature was assigned to the partial deactivation of the active species when the temperature was elevated. The substance of the oligomeric proportions was stochastically changed, implying that the reaction temperature did not effectively control the rate of chain propagation to β-hydrogen elimination. A similar influence of temperature on oligomer distribution using nickel-based systems has previously been reported by our group [23,27,28].

Thirdly, the optimized condition with the Al/Ni molar ratio equal to 500 at 40 °C was used to choose the best run time. The results revealed that the highest activity of 5.99 × 10^6^ g mol^−1^ (Ni) h^−1^ was observed at 15 min, which disclosed that this oligomerization did not have induction time. It was uncommon to observe longer oligomers produced in a shorter period; the portion of C_≥8_ within 15 min (entry 12, Table 2) was more than those in 30 min (entry 9, Table 2), which was more than those in 45 min (entry 13, Table 2), and more than those in 60 min (entry 14, Table 2); such phenomena could be interpreted as the regenerated active species producing shorter oligomers. To have less error caused by operating period, so we finally selected 30 min as the optimized condition.

Under the optimum condition selected by the catalytic system of **Ni1**/EASC, all pre-catalysts **Ni1**–**Ni5** were investigated for ethylene oligomerization in order to elucidate the influence of the ligand structure. The results are summarized in Table 2 (entries 9 and 15–18). Although **Ni3** [2,6-di(iPr)] did not show much activity, other nickel complexes exhibited good activity results, especially **Ni2**, which showed the highest activity among all five catalysts at 4.06 × 10^6^ g mol^−1^ (Ni) h^−1^. Getting rid of the inactive **Ni3**, the other nickel pro-catalysts exhibited high activities and varied in the order: **Ni2** [2,6-di(Et)] > **Ni1** [2,6-di(Me)], **Ni5** [2,6-di(Et)-4-(Me)] > **Ni4** [2,4,6-tri(Me)]. The catalytic properties were affected by both the steric and electronic effects of the substituent of the ligands. It is possible that the groups with greater bulkiness at the *ortho* position of the N-imino aryl ring may protect the active sites from deactivation. The bulkier the R^1^ is, the higher the activity was observed. For example, the activity of **Ni2** (4.06 × 10^6^ g mol^−1^ (Ni) h^−1^) is greater than that of **Ni1** (3.34 × 10^6^ g mol^−1^ (Ni) h^−1^), as well as the similar trend in **Ni5** (3.90 × 10^6^ g mol^−1^ (Ni) h^−1^) exceeded that of **Ni3** (3.03 × 10^6^ g mol^−1^ (Ni) h^−1^). Furthermore, the presence of the additional electron-donating methyl group at 4-position of the aryl ring enhanced the solubility, which can be helpful to activate the complex. However, the existence of methyl group at *para* position resulted in a reduction in activity, due to the lower net charge at the nickel center. So, **Ni2** > **Ni5** and **Ni1** > **Ni4** can be reasonably explained [29,30,31]. In comparison with nickel, iron and cobalt analogs bearing 2-(1-aryliminoethylidene) quinoline [21], which have structures similar to the pre-catalysts examined here, the current series of nickel pre-catalysts showed nearly ten times higher activity for ethylene oligomerization due to the bulkier propyl group attached to the imide carbon, which might stabilize the catalytic center. Thus, these similar series of pre-catalysts with nickel centers are of greater activity than the iron and cobalt ones.

#### 2.2.2. Ethylene Oligomerization in Presence of MMAO

In a similar procedure, the catalytic system using MMAO as a co-catalyst was studied systematically, and the results are summarized in Table 3. When the Al/Ni molar ratio was fixed at 2500, the **Ni1**/MMAO system exhibited the best activity (1.71 × 10^6^ g mol^−1^ (Ni) h^−1^), and oligomerization activity decreased gradually when the Al/Ni molar ratio were raised or lowered (entries 1–5, Table 3). Under the optimum Al/Ni molar ratio, the activities initially increased and then gradually decreased when the temperature was elevated from 20 °C to 60 °C, with the highest activity observed at 1.75 × 10^6^ g mol^−1^ (Ni) h^−1^ at 40 °C (entries 3 and 6–9, Table 3). This phenomenon is consistent with the decomposition of catalysts and a lower absorption of ethylene in the solution at higher temperatures. Differing from the **Ni1**/EASC system, when MMAO used as a co-catalyst the active life of the pre-catalyst was shorter as the activity decreased sharply with longer run times (entries 7 and 10–12, Table 3).

The influence of the ligand properties of the catalyst on oligomerization activities and their oligomer distributions with MMAO as co-catalyst was investigated under the optimum reaction conditions of Al/Ni molar ratio at 2500:1 at 40 °C with 30 min run time. Same as the EASC co-catalyst system, the activity order of **Ni2** > **Ni1**, **Ni5** > **Ni4** was also observed using MMAO as the co-catalyst. Though the catalytic activity of the Ni/MMAO system was lower than that of the Ni/EASC system, more ethylene trimer was obtained, which exhibited a good selectivity in producing hexenes. Of course, this result greatly inspired us to design other catalysts with good selectivity as well as guided us toward the co-catalyst chosen in further studies. Unfortunately, the mechanism resulting in ethylene trimerization is still under exploration.

## 3. Materials and Methods

### 3.1. General Considerations

All manipulations involving air and moisture-sensitive compounds were carried out under an atmosphere of purified nitrogen using standard Schlenk. Toluene was dried over sodium metal and distilled under nitrogen. Methylaluminoxane (MAO, 1.46 M solution in toluene) and modified methylaluminoxane (MMAO, 1.93 M in heptane) were purchased from Akzo Nobel Corp. (Nanjing, China). Diethylaluminium chloride (Et_2_AlCl, 1.17 M in hexane) and ethylaluminium sesquichloride (EASC, 0.87 M in hexane), triethylaluminium (Et_3_Al, 2.00 M in heptane) and other reagents were purchased from Acros Chemicals (Beijing, China). (DME)NiBr_2_ was synthesized by the reaction of 1,2-dimethoxyethane with anhydrous nickel(II) bromide. FT-IR spectra were recorded on a Perkine-Elmer System 2000 FT-IR spectrometer (Shanghai, China). Elemental analysis was carried out using a Flash EA 1112 microanalyzer (Beijing, China). ^1^H- and ^13^C-NMR spectra were recorded on a Bruker DMX 400 MHz instrument (Beijing, China) at ambient temperature using tetramethylsilane (TMS) as an internal standard. GC analyses were performed with a Varian CP-3800 gas chromatograph (Beijing, China) equipped with a flame ionization detector and a 30 m (0.2 mm i.d., 0.25 mm film thickness) CP-Sil 5 CB column.

### 3.2. Synthesis and Characterization of Ligands

*N-(2,2-Dimethyl-1-(quinolin-2-yl)propylidene)-2,6-dimethylbenzenamine* (**L1**). Similar to our previously work, a mixture of tert-butyl 2-quinolyl ketone (0.64 g, 3 mmol), 2,6-dimethylaniline (0.52 g, 3 mmol) and a catalytic amount of *p*-toluenesulfonic acid in tetraethyl orthosilicate (100 mL) was refluxed for 24 h. The solvent was rotary evaporated and the crude product was purified by column chromatography on silica gel (500:5:1 (*v*/*v*/*v*) petroleum ether/ethyl acetate/triethylamine) to give the product as a yellow solid in 63% yield. IR (KBr, cm^−1^): 2963 (m), 2866 (w), 1652 (s), 1589 (m), 1489 (m), 1462 (s), 1357 (m), 1083 (w), 986 (s), 829 (s), 762 (s). ^1^H-NMR (CDCl_3_, 400 MHz, TMS): 8.05 (d, *J* = 8.8 Hz, 1H, quinoline), 7.83 (d, *J* = 8.4 Hz, 1H, quinoline), 7.66 (t, *J* = 7.0 Hz, 2H, quinoline), 7.46 (t, *J* = 7.2 Hz, 1H, quinoline), 6.87 (d, *J* = 8.4 Hz, 1H, quinoline), 6.75 (d, *J* = 7.2 Hz, 2H, *m*-Ar), 6.63 (t, *J* = 7.4 Hz, 1H, *p*-Ar), 2.16 (s, 6H, -CH_3_), 1.46 (s, 9H, -CH_3_). ^13^C-NMR (CDCl_3_, 100 MHz, TMS): 177.4, 157.1, 148.2, 147.2, 135.0, 129.9, 129.5, 127.7, 127.6, 127.1, 126.8, 126.0, 122.6, 119.3, 40.9, 29.2, 18.6. Anal. Calc. for C_22_H_24_N_2_ (316.44): C, 83.50; H, 7.58; N, 8.85%. Found: C, 83.34; H, 7.74; N, 8.69%.

*N-(2,2-Dimethyl-1-(quinolin-2-yl)propylidene)-2,6-diethylbenzenamine* (**L2**). In a manner similar to that described for **L1**, **L2** was prepared as a yellow solid in 67% yield. IR (KBr, cm^−1^): 2965 (m), 2872 (m), 1644 (s), 1593 (s), 1499 (m), 1454 (m), 1364 (w), 1041 (s), 830 (s), 755 (s). ^1^H-NMR (CDCl_3_, 400 MHz, TMS): 8.06 (d, *J* = 8.4 Hz, 1H, quinoline), 7.81 (d, *J* = 8.4 Hz, 1H, quinoline), 7.68–7.64 (m, 2H, quinoline), 7.49–7.45 (m, 1H, quinoline), 6.88 (d, *J* = 8.4 Hz, 1H, quinoline), 6.83 (d, *J* = 7.2 Hz, 2H, *m*-Ar), 6.77–6.74 (m, 1H, *p*-Ar), 2.76–2.67 (m, 2H, -CH_2_-), 2.45–2.37 (m, 2H, -CH_2_-), 1.48 (s, 9H, -CH_3_), 1.21 (t, *J* = 7.6 Hz, 6H, -CH_3_). ^13^C-NMR (CDCl_3_, 100 MHz, TMS): 176.2, 156.8, 147.2, 147.1, 134.8, 131.4, 129.8, 129.3, 127.5, 126.9, 126.6, 125.2, 122.7, 119.3, 40.9, 29.0, 24.6, 13.4. Anal. Calc. for C_24_H_28_N_2_ (344.50): C, 83.67; H, 8.13; N, 8.13%. Found: C, 83.87; H, 8.34; N, 8.01%.

*N-(2,2-Dimethyl-1-(quinolin-2-yl)propylidene)-2,6-diisopropylbenzenamine* (**L3**). In a manner similar to that described for **L1**, **L3** was prepared as a yellow solid in 54% yield. IR (KBr, cm^−1^): 2959 (m), 2920 (m), 1636 (m), 1590 (w), 1499 (w), 1463 (m), 1358 (m), 1040 (s), 1001 (m), 825 (s), 752 (s). ^1^H-NMR (CDCl_3_, 400 MHz, TMS): 8.01 (d, *J* = 8.8 Hz, 1H, quinoline), 7.81 (d, *J* = 8.8 Hz, 1H, quinoline), 7.65–7.62 (m, 2H, quinoline), 7.45 (t, *J* = 7.6 Hz, 1H, quinoline), 6.84–6.82 (m, 3H), 6.79–6.75 (m, 1 H, *p*-Ar), 3.16–3.09 (m, 2H, -CH-), 1.44 (s, 9H, -CH_3_), 1.14 (d, *J* = 6.4 Hz, 6H, -CH_3_), 1.05 (d, *J* = 6.8 Hz, 6H, -CH_3_). ^13^C-NMR (CDCl_3_, 100 MHz, TMS): 175.5, 156.8, 147.1, 145.9, 135.7, 134.6, 129.7, 129.3, 127.4, 126.8, 126.5, 122.7, 122.1, 119.3, 40.8, 29.0, 28.2, 23.6, 21.6. Anal. Calc. for C_26_H_32_N_2_ (372.56): C, 83.82; H, 8.60; N, 7.52%. Found: C, 83.65; H, 8.81; N, 7.58%.

*N-(2,2-Dimethyl-1-(quinolin-2-yl)propylidene)mesitylamine* (**L4**). In a manner similar to that described for **L1**, **L4** was prepared as a yellow solid in 54% yield. IR (KBr, cm^−1^): 2965 (m), 2915 (m), 1680 (w), 1633 (s), 1594 (m), 1499 (m), 1469 (s), 1358 (m), 1000 (m), 827 (s), 748 (s). ^1^H-NMR (CDCl_3_, 400 MHz, TMS): 8.06 (d, *J* = 8.8 Hz, 1H, quinoline), 7.83 (d, *J* = 8.8 Hz, 1H, quinoline), 7.68–7.65 (m, 2H, quinoline), 7.47 (t, *J* = 7.6 Hz, 1H, quinoline), 6.86 (d, *J* = 8.4 Hz, 1H, quinoline), 6.56 (s, 2H, *m*-Ar), 2.11 (s, 6H, -CH_3_), 2.05 (s, 3H, -CH_3_), 1.44 (s, 9H, -CH_3_). ^13^C-NMR (CDCl_3_, 100 MHz, TMS): 177.3, 157.2, 147.1, 145.5, 134.9, 131.5, 129.8, 129.3, 128.1, 127.5, 126.9, 125.6, 119.3, 40.8, 29.0, 20.6, 18.4. Anal. Calc. for C_23_H_26_N_2_ (330.48): C, 83.58; H, 7.87; N, 8.47%. Found: C, 83.34; H, 7.69; N, 8.58%.

*N-(2,2-Dimethyl-1-(quinolin-2-yl)propylidene)-2,6-diethyl-4-methylbenzenamine* (**L5**). In a manner similar to that described for **L1**, **L5** was prepared as a yellow solid in 37% yield. IR (KBr, cm^−1^): 2975 (w), 1683 (w), 1642 (s), 1594 (m), 1500 (m), 1462 (m), 1142 (m), 1038 (s), 818 (s), 799 (s). ^1^H-NMR (CDCl_3_, 400 MHz, TMS): 8.04 (d, *J* = 8.8 Hz, 1H, quinoline), 7.81 (d, *J* = 8.8 Hz, 1H, quinoline), 7.67–7.64 (m, 2H, quinoline), 7.46 (t, *J* = 7.6 Hz, 1H, quinoline), 6.85 (d, *J* = 8.4 Hz, 1H, quinoline), 6.60 (s, 2H, *m*-Ar), 2.69–2.60 (m, 2H, -CH_2_-), 2.35–2.28 (m, 2H, -CH_2_-), 2.10 (s, 3H, -CH_3_), 1.43 (s, 9H, -CH_3_), 1.15 (t, *J* = 7.4 Hz, 6H, -CH_3_). ^13^C-NMR (CDCl_3_, 100 MHz, TMS): 175.2, 156.0, 146.0, 143.5, 133.7, 130.5, 130.1, 128.7, 128.1, 126.4, 125.8, 125.4, 124.8, 118.4, 39.8, 27.9, 23.5, 19.8, 12.4. Anal. Calc. for C_25_H_30_N_2_ (358.53): C, 83.74; H, 8.37; N, 7.81%. Found: C, 83.52; H, 8.54; N, 7.62%.

### 3.3. Synthesis and Characterization of Nickel Complexes

*N-(2,2-Dimethyl-1-(quinolin-2-yl)propylidene)-2,6-dimethylbenzenaminonickel dichloride* (**Ni1**). To add the ligand *N*-(2,2-dimethyl-1-(quinolin-2-yl)propylidene)-2,6-dimethyl-benzenamine (0.32 g, 1 mmol) in a mixed solution of dichloromethane (5 mL) and ethanol (5 mL), (DME)NiBr_2_ (0.28 g, 0.9 mmol) was added. The mixture was stirred at room temperature for 24 h to afford a brown precipitate from the reaction mixture. The resulted precipitate was filtered and washed with diethyl ether (3 × 5 mL) to afford a brown powder (0.28 g, 0.52 mmol) in 59% yield. IR (KBr, cm^−1^): 2981 (w), 2917 (w), 1619 (w), 1591 (s), 1508 (m), 1459 (m), 1173 (s), 1060 (w), 958 (m), 866 (w), 833 (s), 753 (s). Anal. Calc. for C_22_H_24_Br_2_N_2_Ni (534.94): C, 49.39; H, 4.49; N, 5.23%. Found: C, 49.68; H, 4.67; N, 5.11%.

*N-(2,2-Dimethyl-1-(quinolin-2-yl)propylidene)-2,6-diethylbenzenaminonickel dichloride* (**Ni2**). Using a similar procedure as that described for the synthesis of **Ni1**, **Ni2** was obtained in 63% yield. IR (KBr, cm^−1^): 2972 (m), 1617 (w), 1505 (w), 1454 (m), 1383 (w), 1344 (w), 1167 (m), 1064 (m), 1006 (m), 832 (w), 790 (m), 757 (s), 722 (m). Anal. Calc. for C_24_H_28_Br_2_N_2_Ni (562.99): C, 51.20; H, 4.97; N, 4.97%. Found: C, 51.42; H, 5.12; N, 4.76%.

*N-(2,2-Dimethyl-1-(quinolin-2-yl)propylidene)-2,6-diisopropylbenzenaminonickel dichloride* (**Ni3**). Using a similar procedure as that described for the synthesis of **Ni1**, **Ni3** was obtained in 63% yield. IR (KBr, cm^−1^): 2947 (m), 1621 (w), 1456 (w), 1388(w), 1351 (s), 1094 (s), 1001 (s), 800 (w). Anal. Calc. for C_26_H_32_Br_2_N_2_Ni (591.06): C, 52.83; H, 5.41; N, 4.74%. Found: C, 52.98; H, 5.72; N, 4.58%.

*N-(2,2-Dimethyl-1-(quinolin-2-yl)propylidene)mesitylaminonickel dichloride* (**Ni4**). Using a similar procedure as that described for the synthesis of **Ni1**, **Ni4** was obtained in 63% yield. IR (KBr, cm^−1^): 2912 (m), 1629 (w), 1590 (s), 1506 (m), 1463 (m), 1385 (w), 1140 (w), 1004 (m), 960 (w), 833 (m), 760 (s). Anal. Calc. for C_23_H_26_Br_2_N_2_Ni (548.98): C, 50.32; H, 4.74; N, 5.10%. Found: C, 50.20; H, 4.98; N, 4.89%.

*N-(2,2-Dimethyl-1-(quinolin-2-yl)propylidene)-2,6-diethyl-4-methylbenzenaminonickel dichloride* (**Ni5**). Using a similar procedure as that described for the synthesis of **Ni1**, **Ni5** was obtained in 63% yield. IR (KBr, cm^−1^): 2974 (m), 1613 (w), 1591 (m), 1508 (m), 1456 (s), 1387 (m), 1134 (w), 1007 (m), 955 (w), 836 (s), 763 (s). Anal. Calc. for C_25_H_30_Br_2_N_2_Ni (577.03): C, 52.03; H, 5.20; N, 4.85%. Found: C, 52.26; H, 5.31; N, 4.66%.

### 3.4. General Procedure for Ethylene Oligomerization

Ethylene oligomerization at 5 atm ethylene pressure was performed in a stainless steel autoclave (250 mL capacity) equipped with gas ballast through a solenoid clave for the continuous feeding of ethylene at a constant pressure. Twenty milliliters of toluene was added into the autoclave under ethylene atmosphere and the catalyst precursor was dissolved in 30 mL toluene in a Schlenk tube, stirred with a magnetic stirrer and injected into the reactor. With the desired amount of EASC or MMAO, 50 mL toluene (total volume was 100 mL) was added. When the reaction temperature had been reached, ethylene at the desired pressure was introduced to start the reaction. After stirring for the desired period of time, the reaction was stopped and about 2 mL of the reaction solution was collected and terminated by the addition of 4 mL 10% aqueous hydrogen chloride. The organic layer was collected and analyzed by gas chromatography (GC) to determine the composition and mass distribution of the oligomers.

## 4. Conclusions

A series of *N*-(2,2-dimethyl-1-(quinolin-2-yl)propylidene) aryliminonickel bromides have been synthesized and characterized. Four of the nickel complexes performed good activities for ethylene oligomerization, and both the variation of the substituents of the ligands and the reaction parameters affected the catalytic behaviors. The catalytic activities decreased on changing the reaction temperature to either lower or higher that the optimum temperature, consistent with the changes in run time. The Ni/EASC system showed high catalytic activity up to 4.06 × 10^6^ g mol^−1^ (Ni) h^−1^, while the Ni/MMAO system exhibited good characterization of ethylene trimerization. Compared with our previous 2-(1-aryliminoethylidene) quinolylnickel precatalysts [21], the *gem*-dimethylated title nickel complexes displayed higher activities as well as more hexenes. In summary, this illustrates the importance of finely tailoring the substituents affecting catalytic performances of metal complexes as well as the resultant oligomers.

## Figures and Tables

**Table 1:** Effects of co-catalysts on ethylene reactivity ^a^.

Entry	Co-Cat	Al/Ni	Activity ^b^	Oligomer Distribution ^c^ (%)		
				C_4_/ΣC	C_6_/ΣC	C_≥8_/ΣC
1	Et_2_AlCl	400	1.07	55.0	42.1	2.9
2	EASC	400	1.33	28.9	35.5	35.6
3	MAO	1500	0.38	73.4	9.1	17.5
4	MMAO	1500	1.25	9.1	87.1	3.8
5	Et_3_Al	400	0.05	100		

^a^ *Reaction conditions*: 3 μmol **Ni1**, 30 min, 30 °C, 5 atm ethylene, 100 mL toluene; ^b^ 10^6^ g mol^−1^ (Ni) h^−1^; ^c^ Determined by GC. ΣC denotes the total amount of oligomers.

**Table 2:** Ethylene catalytic activity with **Ni1**–**Ni5**/EASC ^a^.

Entry	Cat.	Al/Ni	*t* (min)	T (°C)	Activity ^b^	Oligomer Distribution ^c^ (%)		
						C_4_/ΣC	C_6_/ΣC	C_≥8_/ΣC
1	**Ni1**	200	30	30	0.64	31.1	45.8	23.1
2	**Ni1**	300	30	30	0.81	33.2	49.0	17.8
3	**Ni1**	400	30	30	1.33	28.9	35.5	35.6
4	**Ni1**	500	30	30	2.17	21.4	24.7	53.9
5	**Ni1**	600	30	30	1.81	10.7	24.6	64.7
6	**Ni1**	700	30	30	1.52	9.91	26.4	63.7
7	**Ni1**	800	30	30	1.11	8.65	19.4	72.0
8	**Ni1**	500	30	20	1.38	29.5	22.6	47.9
9	**Ni1**	500	30	40	3.34	15.7	22.9	61.4
10	**Ni1**	500	30	50	2.78	10.2	18.8	71.0
11	**Ni1**	500	30	60	2.49	8.20	15.1	76.7
12	**Ni1**	500	15	40	5.99	16.8	18.1	65.1
13	**Ni1**	500	45	40	3.15	14.7	25.6	59.7
14	**Ni1**	500	60	40	3.01	16.0	26.3	57.7
15	**Ni2**	500	30	40	4.06	15.1	16.3	68.6
16	**Ni3**	500	30	40	trace			
17	**Ni4**	500	30	40	3.03	16.5	20.1	63.4
18	**Ni5**	500	30	40	3.90	16.8	18.8	64.4
19 ^d^	**Ni1**	500	30	40	3.91	18.7	26.0	55.3

^a^ *Reaction conditions*: 3 μmol **Ni1**, 5 atm ethylene, 100 mL toluene; ^b^ 10^6^ g mol^−1^ (Ni) h^−1^; ^c^ Determined by GC. ΣC denotes the total amount of oligomers; ^d^ 10 atm.

**Table 3:** Ethylene catalytic activity with **Ni1**–**Ni5**/MMAO ^a^.

Entry	Cat.	Al/Ni	*t* (min)	T (°C)	Activity ^b^	Oligomer Distribution ^c^ (%)		
						C_4_/ΣC	C_6_/ΣC	C_≥8_/ΣC
1	**Ni1**	1000	30	30	1.10	9.1	80.8	10.1
2	**Ni1**	2000	30	30	1.45	8.9	78.1	13.0
3	**Ni1**	2500	30	30	1.71	7.6	75.7	16.7
4	**Ni1**	3000	30	30	1.63	6.7	71.4	21.9
5	**Ni1**	3500	30	30	1.54	9.0	73.1	17.9
6	**Ni1**	2500	30	20	1.52	9.7	81.3	9.0
7	**Ni1**	2500	30	40	1.75	7.8	88.4	3.8
8	**Ni1**	2500	30	50	1.67	6.8	88.9	4.3
9	**Ni1**	2500	30	60	1.49	5.2	90.0	4.8
10	**Ni1**	2500	15	40	3.18	8.8	88.1	3.1
11	**Ni1**	2500	45	40	0.89	6.7	88.9	4.4
12	**Ni1**	2500	60	40	0.68	6.0	90.5	3.5
13	**Ni2**	2500	30	40	1.81	7.5	86.9	5.6
14	**Ni4**	2500	30	40	1.50	5.9	89.7	4.4
15	**Ni5**	2500	30	40	1.65	8.1	83.4	8.5
16 ^d^	**Ni1**	2500	30	40	1.91	8.7	89.0	2.3

^a^ *Reaction conditions*: 3 μmol **Ni1**, 5 atm ethylene, 100 mL toluene; ^b^ 10^6^ g mol^−1^ (Ni) h^−1^; ^c^ Determined by GC. ΣC denotes the total amount of oligomers; ^d^ 10 atm.
